# Post abortion care quality status in health facilities of Guraghe zone, Ethiopia

**DOI:** 10.1186/1742-4755-10-35

**Published:** 2013-07-23

**Authors:** Gezahegn Tesfaye, Lemessa Oljira

**Affiliations:** 1 Department of Public Health, College of Health and Medical sciences, Haramaya University, Harar, Ethiopia

**Keywords:** Quality, Post abortion care, Abortion, Health facility

## Abstract

**Background:**

Unsafe abortion in the developing world accounts for 13% of all maternal deaths. Ethiopia is one of the developing countries with the highest maternal mortality ratio (673 per 100,000 live births) in the world. Unsafe abortion was estimated to account for 32% of all maternal deaths in Ethiopia.

**Objective:**

To assess post abortion care quality status in health facilities of Guraghe zone.

**Methods:**

A facility based cross-sectional study design with both quantitative and qualitative methods was conducted. Patient interview, direct service observation, provider self administered questionnaire and inventory of equipment and supplies were used for the assessment. Six health centers, two hospitals and 422 post-abortion patients were included in the study.

**Results:**

Patient-provider interaction was generally satisfactory from the patient’s perspective. The majority of the respondents (93.5%) said that they were treated with politeness and respect. More than half 226(56.5%) of the clients have received post abortion family planning. Overall, 83.5% of the patients were satisfied with the services. Those who said waiting time was long were less satisfied and unemployed women were more satisfied than others.

**Conclusion:**

The study has revealed several improvements as well as problems in the provision of post-abortion care service in the studied health facilities.

## Background

World Health Organization (WHO) defines unsafe abortion as a procedure for terminating an unintended pregnancy carried out either by persons lacking the necessary skills or in an environment that does not conform to minimal medical standards, or both. Unsafe abortion and deaths due to complications of unsafe abortion continue to afflict the lives of many women, mostly in developing countries. Unsafe abortion is the cause of serious complications and disability for millions of women each year and is a prominent cause of maternal death [1]. An estimated 30, 000 deaths due to unsafe abortion have occurred in the year 2000 in Africa which is over 40% of the total deaths due to unsafe abortion globally [2].

Among the causes of maternal mortality in developing countries, unsafe abortion accounts for 13% of maternal deaths. Ethiopia is one of the developing countries with the highest maternal mortality ratio in the world. It is estimated to be 673 per 100,000 live births. Unsafe abortion was estimated to account for 32% of all maternal deaths in Ethiopia [3].

Unsafe abortion was recognized as a major public health problem at the International Conference held on Population and Development (ICPD) in 1994. The participants called for prompt, high quality and sympathetic medical services to treat the complications of unsafe abortion. Additionally, the ICPD emphasized the importance of post-abortion counseling and family planning services as part of a comprehensive package of post-abortion care to promote reproductive health and prevent repeated abortions [4].

Comprehensive post abortion care was identified as an important intervention to treat complications resulting from miscarriage and unsafe abortion, reduce the incidence of repeat unplanned pregnancy, and decrease the incidence of repeat abortion [5]. In Ethiopia problems related to abortion were neglected and access to quality post-abortion care was very limited [6-8].

Women can have an early pregnancy loss from either a miscarriage or self-induced abortion. Both can be life threatening from hemorrhage, infection, shock, and blood clotting problems. One surprising finding in most studies is the lack of quality post abortion care and women are treated without any pain relief and with risky methods such as sharp curettes. Even more disconcerting was that after a pregnancy loss, women were not given family planning information or supplies to space their next pregnancy [9]. Restrictive national laws, lack of access to safe abortion and lack of quality post-abortion car have led to the premature death of millions of mothers [10,11].

So far studies done in Ethiopia have addressed mostly the magnitude, cause, setting and distribution of abortion. In addition, while several studies have examined reproductive health service utilization in both developed and developing countries there is only scant information available about quality of post abortion care in health facilities from Ethiopia except a few facility based studies on quality of PAC in the country. This study is believed to fill such an information gap and enable to identify areas of service improvement.

Therefore the objective of this study was to assess the post abortion care quality status in health facilities of Guraghe zone, Ethiopia.

## Methods and materials

### Study setting

The study was conducted in Guraghe zone. In the zone there were 26 health centers and three hospital (one public and two non governmental). According to the zonal health bureau eleven health facilities have initiated PAC service (two hospitals and nine health centers). The study was conducted from January to March, 2010. In the selected health facilities the qualitative study was done by direct service delivery observation and inventorying using checklist of supplies, equipments and medications.

### Study design

A facility based cross-sectional study involving both quantitative and qualitative methods was conducted. The study has measured the following aspects of quality of PAC; client satisfaction, providers technical competence, availability of equipment and supplies.

### Study population

Women who sought PAC services and health care providers directly involved in PAC services in all health facilities that provide PAC service (six health centers and two hospitals) during the study period. Health facilities which were difficult to reach or inaccessible due to logistical reasons were excluded from the study. Also, those patients who have hearing problem, mentally disabled, patients who declined to participate, the severely ill, patients who left against medical advice or had a gestational age greater than 28 weeks were excluded from the study.

### Sample size determination

The sample size was determined using the following assumptions (Level of significance of the population was taken to be 95%, Zα/2 = 1.96). A 5% level of precision (d = 0.05) and 50% proportion of women satisfied with PAC service (P = 0.5). Therefore the total sample size for this study was 422 women seeking PAC service including 10% none respondent rate. All health care providers who were directly involved in PAC service at the studied health facilities during the study period were included.

### Sampling technique

All post abortion patients consecutively discharged from the selected health facilities were included in the study until the required number of cases reached. This was done through a quota sampling technique which was a recommended method to study abortion in a facility setting [2].

### Data collection method

Client exit interviewing was done by female staff nurses working in another department in the same facility. Female staff nurses were preferred to conduct the interviews because our study participants were females and it was highly likely that they provide unbiased information to female interviewers. The data collectors were trained for three days by the principal investigator on the objectives of the study and how to conduct the interview, fill in the questionnaire and handle questions asked by clients. Data from service providers was collected using a self-administered questionnaire in English. Experienced health officers and BSc nurses who have received training on comprehensive PAC conducted the service delivery observation using a check list. They received a one day orientation on methodology of observation. Verbal consent was obtained from the patients and providers before conducting the observation. The observers were in white coats and remained inconspicuous so as not to interfere with routine service provision. The principal investigator used a standardized check list to assess availability of equipments, supplies and medications for post abortion care service in all the studied health facilities.

### Data analysis

Responses were coded and entered using Epi info version 3.5.1. Data were then exported to SPSS version 15 for further analysis. Descriptive statistics and summary measures of the variables were conducted. A crude and adjusted odds ratio with 95% confidence interval from bivariate and multi-variate analyses was used to measure association between dependent and independent variables. Data were also obtained from inventorying and observation check list. They were extracted, organized, presented and pertinent information was examined in relation to the quantitative results in which it was triangulated with the other quantitative results and presented separately.

### Data quality control

Standardized observation and an inventorying check list were used. A questionnaire for client exit interview was adapted from similar study in Ethiopia [6] and modified to the study context by reviewing other previous similar studies. Its English version was translated into Amharic and again back to English so as to ensure its consistency. The questionnaire was pretested on 21 clients in one of the health facility other than the selected study facilities and corrections were made on omitted, an answerable or unclear questions accordingly. Data collectors were health professionals who have experience on the area and received proper training on the data collection tool. Five percent of the data was reentered and compared with the already entered data.

### Ethical consideration

Ethical clearance was obtained from Haramaya University institutional research ethics review committee. An official letter of co-operation was also written to the Guraghe zone health bureau. Written informed consent was obtained from each study participant. Confidentiality was maintained by avoiding names and other personal identification information whereas privacy was maintained by conducting the exit interview in a separate room that offered visual and auditory privacy.

### Operational definitions

1. Post abortion patients: are any patient presenting with sign and symptom of abortion and declared by the provider in charge as having an abortion regardless of the cause and type.

2. Providers: refers to health professionals involved in history taking, physical examination, treatment and counseling of post abortion cases.

3. Quality: “Quality of PAC” assessed based on client satisfaction, providers technical competency and set up or facility assessment.

4. Client satisfaction: overall client’s perception toward the PAC services she received.

5. Patient provider interaction: “Provider who possesses good listening skills understanding and cares for the woman in “respectful” way and in a private environment.

6. Technical competence: refers to qualification, training background, skills and experience of providers.

7. Information received: when a post abortion patient received information on family planning, danger signs and need for follow up ‘as much as the patient wanted'.

## Results

### Socio-demographic characteristics

Four hundred twenty two women were identified and interviewed from eight health facilities. Twenty two women refused to participate in the study making the response rate 95%. The age of post-abortion clients ranged from 15 to 41 years with a mean age of 25.3 years (SD _±_ 6.4). A total of 193(48.2%) participants were in the age group between 20 to 29 years. One hundred eighty five (46.2%) were housewives, 134(33.5%) were students and 29(7.2%) were daily laborers. Two hundred twenty seven (56.8%) of them were married and 222(55.5%) attended formal education varying from primary school to tertiary level.

### Reproductive history

Two hundred twenty nine (57.2%) were pregnant at least once before the current pregnancy ended in abortion. The number of children delivered by the women before the current pregnancy was ranged from having no children to eight children. Only 60(17%) gave history of previous abortion. Ninety eight (24.5%) reported that current pregnancy ended in abortion was wanted while the rest 302(75.5%) reported unwanted. Two hundred eighty two (70.5%) knew at least one contraceptive method. A history of use of contraceptives was 243(60.8%). Those who reported their current pregnancy was unwanted had partner pressure 122(40.4%) and negligence to use contraceptives regularly 102(33.8%) as a main reason for the pregnancy. The rest 31(10.3%) were uninformed about contraceptives.

The study showed that the reasons given for resorting to unsafe abortion among post abortion patients were having too close or too many pregnancies 20(40.9%), economic reason 18(36.7%), health reason 3(6.1%), partner pressure 4(8.1%), to complete their education 4(8.1%) (Table 1).

**Table 1:** Showing reasons given for resorting to unsafe abortion among post abortion patients in health facilities of Guraghe Zone, from January to March 2010

**Reasons given**	**Frequency**	**Percent**
Having too close or too many pregnancies	20	40.8%
Economic reason	18	36.7%
Health reason	3	6.1%
Partner pressure	4	8.1%
To complete their education	4	8.1%

### Patient provider interaction

The majority of the respondents 374(93.5%) said they were treated with politeness and respect. Data from service observations also showed that majority 78(95%) of the patients were greeted in a friendly and polite manner and in 70(87.5%) of the patient the service providers were supportively speak to them during the patient provider interaction. Most of the service providers 69(86.3%) did not introduce themselves to patients by name. Providers also gave information on patient’s current illnesses 353(88.3%).

### Information provision

With regard to information provision; 302(75.5%) of the post abortion cases received information on current illness and family planning counseling, and 138(34.5%) of the cases were not told about danger signs that may necessitate revisiting the facilities. Follow-up appointment was given only for 76(19.0%) of the post abortion women.

### Post abortion family planning

When asked on how soon they could become pregnant again if they had sexual intercourse, 98(24.5%) said within two weeks, 123(30.8%) within one month, 110(27.5%) with in three months and 57(14.3%) with in six months while 10(2.5%) of them said after six months.

**Figure 1:**
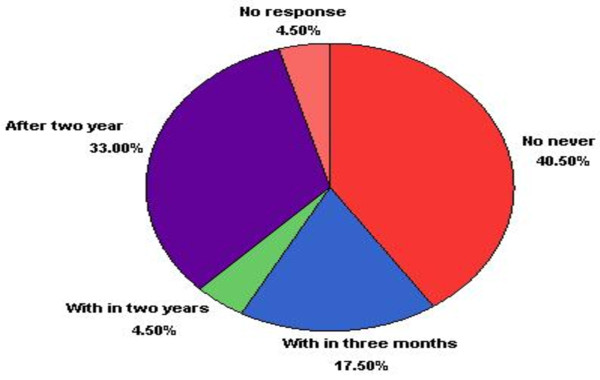
Pregnancy intention of post abortion patients in health facilities of Guraghe Zone, from January to March 2010.

Majority 312(78%) of the post abortion clients have no intention to be pregnant at least within three months. Of which 178(40.5%) revealed that they never want to be pregnant, 132(33%) intended to be pregnant after two years and 18(4.5%) have intended to be pregnant within two years. But a small proportion 70(17.5%) said they intended to be pregnant within three months (Figure 1).

Two hundred twenty six (56.5%) of the post-abortion patients received at least one method of family planning method. In some of the cases, no one raised the issue of contraceptives at all and some said the contraceptive they selected was not available in the facility when mentioning the reason why they were not provided contraceptives.

### Access to service and satisfaction

Ninety six (24%) of the patients responded that the waiting time between arrival and treatment was very long. Fifty six (14%) of patients reported that they were not given pain medication though they had pain during their stay in the health facilities while the rest 257(64.3%) received pain medication and 87(21.7%) had no pain. Overall, 334(83.5%) of the patients expressed that they were satisfied with the services received.

Among those 82(20.5%) patients who have difficulty in locating the service the majority 50(61%) mentioned presence of uncooperative staff as their main reason for their difficulty in getting the service (Table 2).

**Table 2:** Showing reason mentioned by PAC patients for having difficulty in locating or getting the services in health facilities of Guraghe Zone, from January to March 2010

**Reason for difficulty in locating or getting the services**	**Frequency**	**Percent**
Uncooperative staff	50	61
Absence of adequate signs	12	14.6
No enough money	16	19.5
Services were closed	4	4.9
Total	82	100.00

Multivariate analysis (Adjusted for age, marital status, educational status, wanted current pregnancy and pain treatment) showed that statistically significant difference was observed on response to waiting time and occupational status. Those who said that waiting time was long were less satisfied (AOR = 0.230, CI = (0.118, 0.450)) than their counter part. Compared to employed post abortion clients, unemployed women were more likely to be satisfied with the post abortion service delivered (AOR = 2.566, CI = (2.12, 30.983)).

### Technical competence

From the 34 health professionals who received the questionnaire, 9(26.5%) were health officers, 20(58.8%) were nurses and 5(14.7%) were general practitioners who provide services during data collection period. The majority of the nurses, health officers and all general practitioners handled pelvic examination, history taking and operative procedures. Some nurses were involved in taking vital signs, counseling and assisting physicians. Twelve (35.3%), 12(35.3%) and 10(29.4%) had training on STDs counseling, HIV/AIDS counseling and MVA/EVA during their basic education respectively.

Regarding training background 10(29.4%) and 17(50%) providers have taken a refresher or post basic training on FP counseling and method provision and MVA/EVA respectively. Ten (29.4%) of the providers reported FP together with other reproductive health care, followed by FP alone 5(14.7%) and emergency care with FP 4(11.8%) and emergency care with RH 4(11.8%) were not adequately provided. Regarding best place to provide family planning counseling and method 9(26.5%) reported in the MCH center, 10(29.4%) in the recovery ward while 15(44.1%) at both sites. With respect to responsibility to provide the FP service 12(35.3%) responded that it is a responsibility of staff in the MCH while 22(64.7%) reported that it is responsibility of all the staff.

### Inventory

Relevant equipments, supplies and medications required to provide PAC in all the studied health facilities were assessed. The availability of equipments, supplies and medications in the health facilities to fulfill the required basic equipment by the MOH, WHO and international organizations such as IPAS were examined. We have selected list of equipments recommended by WHO (such as water sink), supplies required by MOH (such as glove) and medications required by IPAS (such as from antiseptic). From the equipments, supplies and medications required, the facilities have most of the materials. Except in the two hospitals; items like emergency light source apart from backup generators were absent from most of the studied health centers. Equipments and facilities such as toilet near to PAC rooms, sinks and running water were absent in some of the health facilities. Furthermore vital equipment like ambu bags, oral airways, suction apparatus, oral air ways and oxygen apparatus were absent in most of the unit of service of the health centers and hospitals but they were available in the major operating theaters of the hospitals that were not always near to where PAC provided. Family planning methods were available in adequate amount but not in adequate mix. Especially in one faith-based hospital all types of contraceptive were totally absent except for condoms.

### Direct service observation

From eighty of the cases observed during the pre procedure 27(33.8%) of them had evidence of visual or audible pain during physical examination, and only in 11(13.8%) of them the pain seems adequately controlled. During the procedure 68(85%) of patients were asked if they were in pain, in 61(76.3%) of the cases there was evidence of pain and the pain was not adequately controlled throughout the procedure and only 17(21.3%) were given pain medication.

## Discussion

The study showed that out of 302(75.5%) unwanted pregnancies that resulted in abortion only 49(12.3%) were reported to be interfered. Majority 78% of the participants have no intention of getting pregnant at least in the coming three months. More than half 226(56.5%) of the post abortion cases have received at least one method of contraceptive. Only 138(34.5%) were told to revisit the facility if danger signs happens. Among those 82(20.5%) patients who have difficulty in locating the service the majority 50(61%) mentioned presence of uncooperative staff as their main reason for their difficulty in getting the service. Overall, 334(83.5%) of the patients expressed that they were satisfied with the services received. Short waiting time and unemployment were associated with high satisfaction with the service. Appropriate equipments and supplies needed for providing PAC including MVA equipments were available in all the health facilities.

In this study, out of 302(75.5%) women who reported that the current pregnancy that resulted in abortion was unwanted only 49(12.3%) of them admitted that the abortion was because of interference. WHO classification of abortion puts the group who admitted interference in the certainly induced abortion category where as those who said current pregnancy was unwanted but denied interference in the category of possibly induced abortion [2]. The number of interference with current pregnancy may be higher than stated in the study. Therefore preventing unintended pregnancies and unsafe abortion through improved and expanded family planning services must therefore continue to be a high priority for improving women’s reproductive health in the country.

Post-abortion patients seeking treatment are often under severe emotional stress besides the physical illness they may have. Quickly establishing a good, positive relationship can help ease the anxiety and concern that patients may feel [12]. In contrary to this fact data from service observations in this study showed that majority (86.3%) of the service providers didn’t introduce themselves to the patient as introduction could have led to a more smooth interpersonal relationship.

With regard to information pertaining to complications or danger signs only 138(34.5%) were told to revisit the facility if the danger signs happens is relatively high as compared to a study done in government hospitals of Addis Ababa 21(5.3%) showing an improvement in terms of service provider awareness about the importance of providing this information from time to time but still needs immense attention [6]. Women who wish to terminate their pregnancy should have ready access to reliable information, compassionate counseling and, in parallel, services for the prevention of unintended pregnancy and management of complications [4].

Need for family planning was well reflected as 78% of the interviewees have no intention of getting pregnant at least in the coming three months. This study revealed that 226(56.5%) of the cases received contraceptive method which was essentially one out of every two patients. This is, however, encouraging as compared to a study conducted in Tigray (31%) and public health facilities in Ethiopia (44.7%) of the PAC cases received family-planning services before leaving the facility [8,13]. Family planning program should be intensified to meet the needs of post abortion patients and developments in policy as well as program should intend to improve contraceptive use among post abortion clients through addressing the unmet need for post-abortion family planning services to prevent repeat unplanned pregnancies.

Considering the fact that the illness is almost always life-threatening emergency, attention should be given to ready access to services. To the contrary in this study among those 82(20.5%) patients who have difficulty in locating the service the majority 50(61%) mentioned presence of uncooperative staff as their main reason for their difficulty in getting the service followed by 19.5% because of lack of money which calls for more attention. Due to the nature of unsafe abortion and its complications, health care providers should be ready to respond to it in a very supportive and cooperative way.

The overall client satisfaction level 334(83.5%) was slightly higher than a previous study in Ethiopia [8] where 79.6% of the patients expressed their satisfaction with the services they received and lower than a study in government hospitals in Addis Ababa [6] in that 92.5% of the patients expressed that they were satisfied with the services received. These results imply that the service is doing a reasonably good job from the perspective of the clients in this and other studies. In this study as in previous study [6] short waiting time is associated with high client satisfaction but in the same study unemployment was found to have no association with client satisfaction.

This study has also shown that significant proportions of providers were trained on important aspects of PAC. Appropriate equipments and supplies needed for providing PAC including MVA equipments were available in all the health facilities. This was considerably better than a facility-based assessment of quality of post-abortion care done in Ethiopia where only one-quarter of the health facilities had MVA equipments [7]. These achievements were mainly due to the joint effort by development partners in the health sector in the last twenty years and a result of the current effort to reduce maternal death in Ethiopia.

### Strength and weakness of the study

The strength of the study; the study has considered different assessment techniques such as patients and provider’s perspective, inventory assessment and service observation. This study also has some weaknesses. During the service observation there could be a tendency by service provider to be at their best performance due to the presence of an observer. And the study has only focused on public health facilities and doesn’t give picture of PAC practice in private health facilities.

## Conclusion

The interaction of patients and service providers was satisfactory. However, from a clinical service delivery stand point, important medical information on danger signs, follow-up needs of post abortion clients and care associated pain management were neglected by most of the health professionals. Most of the clients were satisfied with the service provision. Long waiting time was associated with less level of client satisfaction and unemployed women were more satisfied than others. Almost all of the health facilities had basic and appropriate medical equipment and supplies required for providing post abortion services. A majority of the service providers have taken training that is not up to date and focus on general management of PAC clients.

### Possible actions

1. Special attention should be given to information provision regarding danger signs, follow up needs and pain management practice associated with the care should be improved.

2. Methods in reducing waiting time to get the service should be examined.

3. Further large scale study involving private health facilities should be done.

## Competing interests

The authors would like to declare that we have no competing interest in this study.

## Authors’ contributions

GT has conceived of the study, carried out the overall design and execution of the study, performed data collection and statistical analysis and drafted the manuscript. LO has participated in the revision of the design of the study, data collection techniques and helped the statistical analysis. LO co-author of this manuscript. All authors read and approved the final manuscript.
